# Evaluating the effectiveness of mass drug administration on lymphatic filariasis transmission and assessment of post-mass drug administration surveillance in Nigeria’s Federal Capital Territory

**DOI:** 10.1186/s40249-025-01333-5

**Published:** 2025-07-09

**Authors:** Juliana Ajuma Amanyi-Enegela, Joseph Kumbur, Faizah Okunade, Donald Ashikeni, Rinpan Ishaya, Girija Sankar, William Enan Adamani, Moses Aderogba, Louise Makau-Barasa, Achai Emmanuel, Bosede Eunice Ogundipe, Chinwe Okoye, Babar Qureshi

**Affiliations:** 1 National Lymphatic Filariasis Elimination Programme, Neglected Tropical Diseases, Public Health Department, Federal Ministry of Health, Abuja, Nigeria; 2 Department of Public Health, Federal Capital Territory Administration, Abuja, Nigeria; 3 Health and Development Support Programme (HANDS), Jos, Nigeria; 4 CBM Christian Blind Mission Okemesi Crescent Garki 2, Abuja, Nigeria; 5 CBM Christian Blind Mission, Wellington house, Cambridge, UK; 6 The END Fund, New York, USA

**Keywords:** Lymphatic filariasis, Mass drug administration, Transmission, Assessment, Surveillance, Elimination, Nigeria, Sub-Saharan Africa

## Abstract

**Background:**

Nigeria’s Federal Capital Territory (FCT) launched annual mass drug administration (MDA) in its four lymphatic filariasis (LF)-endemic councils in 2011, achieving sustained high coverage and pre-transmission assessment survey success. This study aimed to confirm transmission interruption in Bwari and Gwagwalada and to evaluate post-MDA surveillance efficacy in Abaji and Kuje.

**Methods:**

Transmission Assessment Surveys (TAS) were systematically conducted in four distinct evaluation units (EUs) within the FCT. TAS 1 was carried out in Bwari and Gwagwalada EUs that had recently achieved pre-TAS thresholds indicating potential interruption of transmission, whereas TAS 2 was conducted in Abaji and Kuje EUs, where MDA had been discontinued since 2021 following successful TAS 1 evaluations. Abbott Filarial Test Strips (FTS) were employed to test children aged 6–7 years attending selected schools. Data collection adhered to standardized WHO guidelines, utilizing both paper-based and electronic data-capture tools to enhance accuracy and reduce human error.

**Results:**

A total of 6,448 children participated in surveys across the four EUs, with gender distribution closely balanced (53% male, 47% female). In TAS 1 (Bwari and Gwagwalada), no LF-positive cases were identified well below the WHO-defined critical cutoff of 18 cases. In TAS 2 (Abaji and Kuje), a single LF-positive case was detected in Abaji, still below the critical threshold. Participant refusal rates were minimal, reflecting strong community support and engagement.

**Conclusions:**

The findings provide compelling evidence of significant progress toward LF elimination in Nigeria’s FCT; however, the single positive case in Abaji underscores the continued importance of vigilant surveillance and integrated vector-management strategies to maintain elimination status and guard against residual transmission.

## Background

Lymphatic filariasis (LF) remains a major public health concern, affecting an estimated 51 million people worldwide and placing approximately 882 million individuals in 44 countries at risk [1]. Caused by the filarial nematodes *Wuchereria bancrofti*, *Brugia malayi*, or *B. timori*, LF is transmitted by mosquitoes, leading to debilitating chronic conditions such as lymphedema, hydrocele, and acute adenolymphangitis [2, 3]. The disease is classified as one of the neglected tropical diseases (NTDs) and disproportionately affects impoverished communities in the tropics and subtropics [4, 5].

The African continent bears the highest collective burden of LF, with 31 of the 44 countries requiring treatment programs located in sub-Saharan Africa alone [5]. Nigeria has the highest prevalence in Africa, where approximately 100 million people in 583 local government areas are considered at risk [4, 6].

**Fig. 1:**
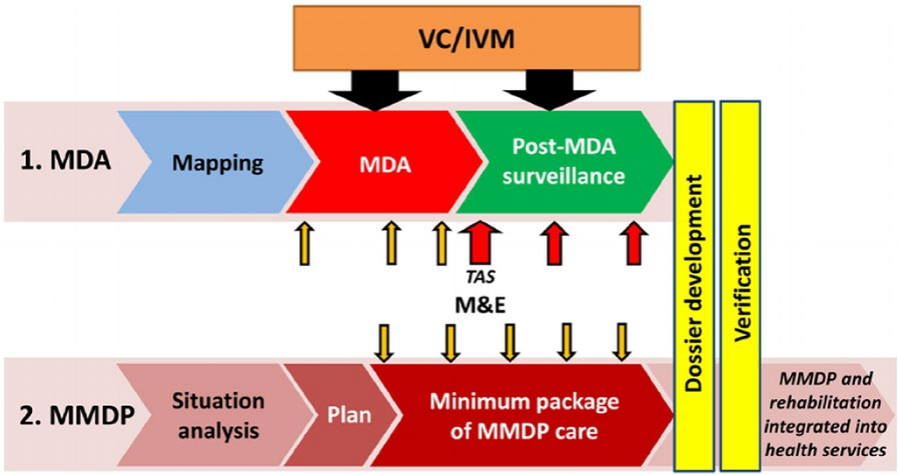
Global Programme to Eliminate Lymphatic Filariasis strategy. Source: [9]. Abbreviations: *MDA* mass drug administration; *MMDP* morbidity management and disability prevention; *VC* vector control;* IVM* integrated vector management

In response to this substantial burden, World Health Organization (WHO) in 1997 formally targeted LF for global elimination [7]. The two-pronged strategies recommended for elimination of LF as a public health problem in endemic areas are: Mass drug administration (MDA) to reduce microfilaria loads and interrupt transmission, and morbidity management and disability prevention (MMDP) to mitigate suffering among those already affected [7–9]. The impact of MDA is assessed through pre-transmission assessment surveys (Pre-TAS) and transmission assessment surveys which confirm if the prevalence of LF in an area has declined to levels at which MDA can be safely stopped [10, 11]. This usually happens after at least five successful rounds of effective MDA coverage (i.e., reaching at least 65% of the total population across all endemic communities). WHO [10] also recommends three TAS in two-year intervals as post-MDA surveillance and to demonstrate sustained interruption of transmission in previously endemic areas. The WHO recommended strategy for LF elimination in endemic areas is shown in Fig. 1. The figure highlights the step-by-step approach recommended for LF elimination in endemic areas.

In Nigeria, implementation of MDA has shown promise in reducing LF transmission, with numerous endemic areas meeting criteria for scaling down interventions following consecutive years of effective coverage.[12]. However, maintaining the gains achieved through MDA requires rigorous post-MDA surveillance to promptly detect any resurgence of transmission and to ensure that momentum toward elimination is not lost [13]. The Federal Capital Territory (FCT), home to Abuja, exemplifies this dual challenge. Baseline surveys conducted between 2008 and 2010 established that four of the six area Councils (Abaji, Bwari, Gwagwalada, and Kuje) were LF-endemic [14]. As a result, annual MDA commenced in these councils in 2011. By 2021, TAS 1 results indicated the interruption of transmission in Abaji and Kuje, leading to the stopping of MDA in those two area councils, while Bwari and Gwagwalada continued annual treatment regimens.

TheTAS aimed to determine whether sustained MDA efforts successfully interrupted LF transmission in Bwari and Gwagwalada after over five consecutive years of intervention and to gauge the performance of post-MDA surveillance in Abaji and Kuje, where drug distribution ceased in 2021.

This paper provides results of TAS 1 which was conducted in 2024 after over five years of MDA in Bwari and Gwagwalada evaluation units (EUs) and highlights the findings of TAS 2 as part of post-MDA surveillance in Abaji and Kuje, where MDA was stopped in 2021. The result of these assessments provides insights and evidence that will inform the next steps for LF elimination strategies within the FCT and other LF-endemic regions in Nigeria and beyond.

## Methods

### Study site and sample size

The transmission assessment survey was conducted in Abuja, Nigeria’s FCT a 7300-km^2^ Guinea-savannah region (≈ 8° N, 7° E) with a tropical savannah climate (April–October rains, November–March dry season; mean annual temperature ≈ 26 °C) [15].

**Fig. 2:**
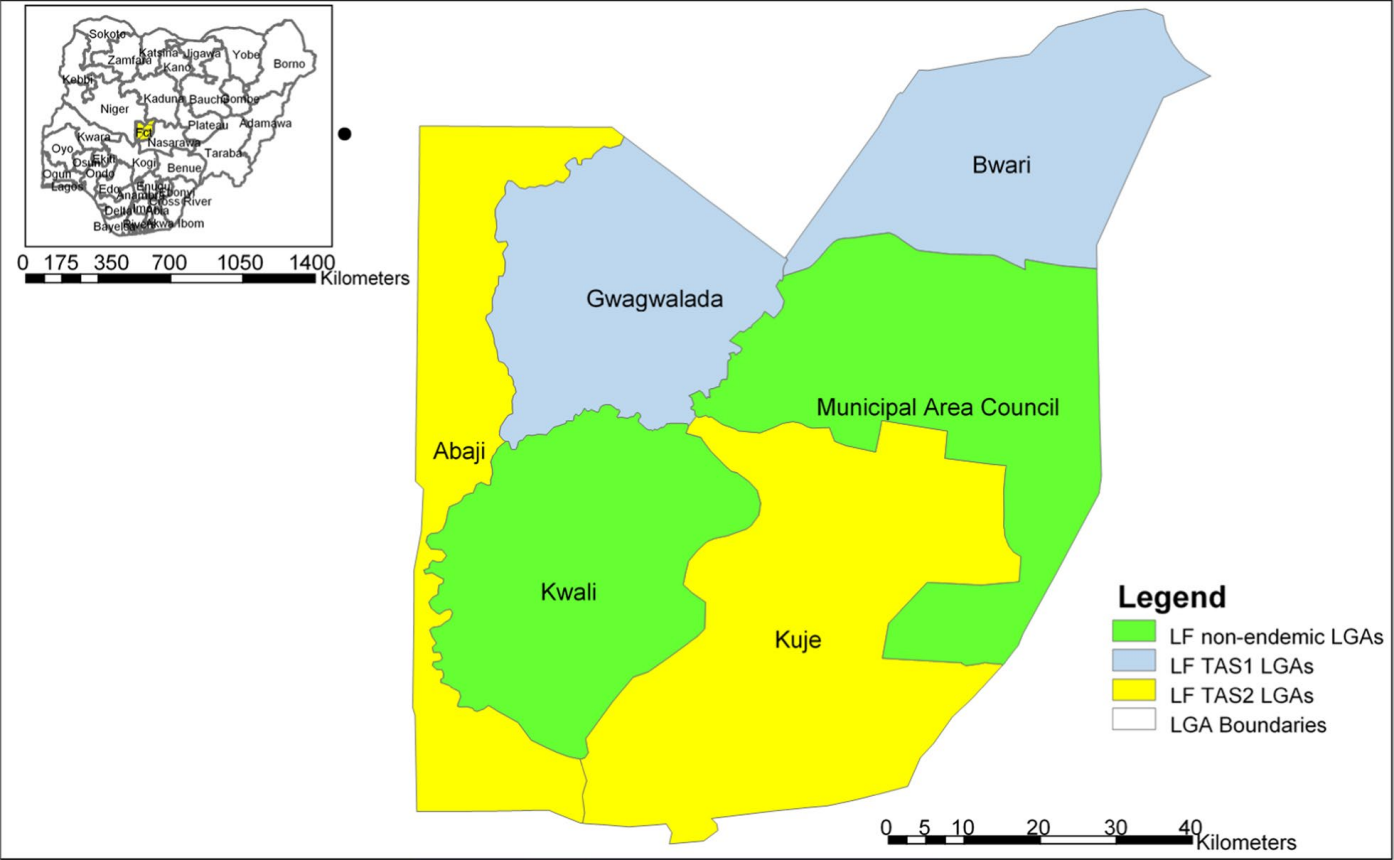
Map of Federal Capital Territory showing the four evaluation units for TAS. Abbreviations: *LF* Lymphatic filariasis; *LGAs* Local government areas; *TAS* Transmission assessment survey

Four lymphatic-filariasis-endemic area councils—Abaji, Bwari, Gwagwalada and Kuje, served as evaluation units. Together they host roughly 1.2 million residents distributed across peri-urban settlements that fringe Abuja city (Bwari, Gwagwalada) and predominantly rural farming communities (Abaji, Kuje) [15]. Health services are delivered through an extensive network of primary-care facilities and community drug-distributor teams that have implemented annual MDA for LF since 2011. The survey was conducted across the four EUs in June 2024. The EUs where the survey was conducted is highlighted in the map of FCT in Fig. 2.

**Fig. 3:**
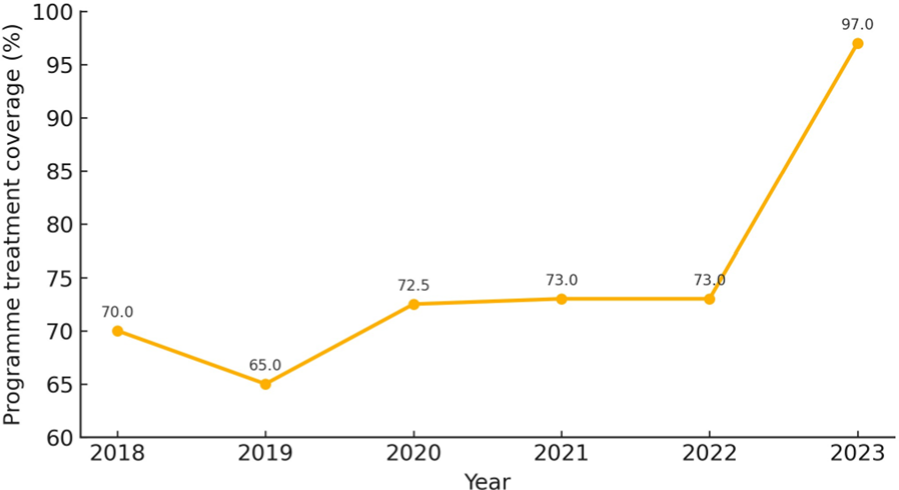
Bwari area council programme treatment coverage showing effective rounds of mass drug administration coverage between 2018–2023

**Fig. 4:**
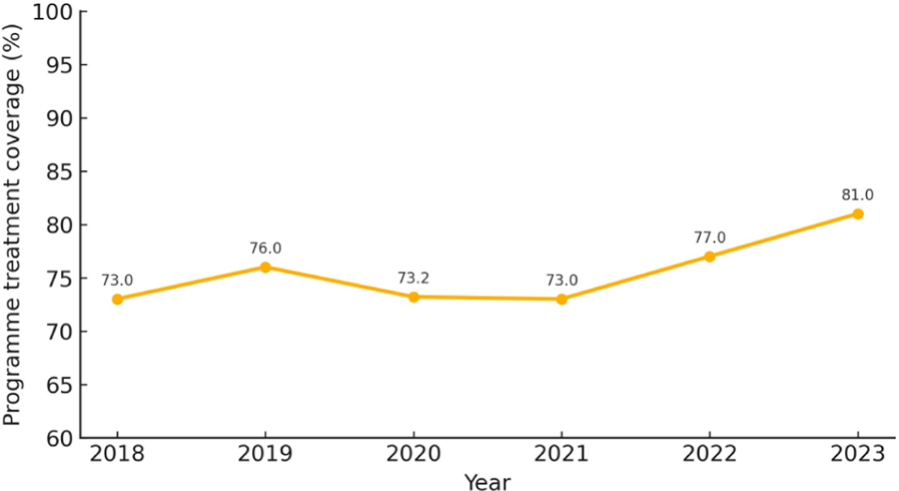
Gwagwalada area council programme treatment coverage showing effective rounds of mass drug administration coverage between 2018 and 2023

TAS 1 was conducted in Bwari and Gwagwalada EU. These EUs achieved five years of effective coverage (65%) as shown in Fig. 3 and 4, and result of pre-TAS conducted in 2023 indicated a suspected interruption of transmission.

### Study site, sample size and sampling technique

TAS 2 was conducted in Abaji and Kuje EUs where MDA was stopped in 2021. Figure 2 shows the location of the EUs where TAS 1 and TAS 2 were conducted.

The survey covered four EUs within the FCT: Abaji, Kuje, Gwagwalada, and Bwari. A cluster sampling strategy was used in all surveyed EUs. Each EU had more than 1,000 pupils and 40 schools, which qualified them for this sampling strategy. The target sample sizes, the target number of schools, and the number of additional schools generated by the Survey Sample Builder (SSB) are shown in Table 1.

**Table 1:** Target sample size and school allocation across four evaluation units, FCT LF transmission assessment survey

S/N	State	Area councils	EU	Target sample size	Target number of schools	Number of extra schools
1	FCT	Abaji	1	1532	30	15
2	FCT	Kuje	2	1532	30	15
3	FCT	Gwagwalada	3	1540	30	15
4	FCT	Bwari	4	1552	30	15

*EU* Evaluation unit; *FCT* Federal Capital Territory; *S/N* Serial number; *LF* Lymphatic filariasis

### Training of survey team

To ensure compliance with WHO guidelines during data collection, survey teams, including team leaders, laboratory scientists and data managers were trained on the standard operating procedures to follow in the field. The training focused on modules from the WHO guide for TAS implementation and the practical use of the Abbott Filarial Test Strip (FTS). Post-training assessments was conducted, and the results revealed a significant improvement in participants' knowledge of LF TAS from 48 to 89%.

### Test procedure

The survey included children aged six and seven who were enrolled in the selected schools. In some instances, children above 7 years were included due to varying ages of children in the classes selected. Children were excluded if they had received LF treatment within the past six months or showed signs of illness, such as fever.

Blood samples of 75 μl were collected from participating pupils using sterile lancets. The samples were tested with FTS devices, and the results were read within 10 min of sample collection. The FTS kits were supplied by Abbott Diagnostics Scarborough Inc., located in Scarborough, Maine, USA. Capillary blood was obtained using BD Microtainer® contact-activated sterile lancets manufactured by Becton, Dickinson & Co., based in Franklin Lakes, New Jersey, USA. Invalid and positive tests were retested to ensure accuracy. Upon completion of the survey, feedback was provided to the head teachers at each participating school.

### Data collection, validation and analysis

Data collection was done using both hard copy and electronic forms and analysed using Microsoft Excel 2016 (Microsoft Corporation, Redmond, WA, USA). The data collection tools captured key indicators such as gender, age, consent to participate, unique identification number, and school code. Android devices were utilized to record the geographic coordinates of the survey sites. Continuous variables (e.g., age) were tested for normality. If normally distributed, data were summarized as mean ± standard deviation (SD); if not, median and interquartile range (IQR) were reported. Categorical data (such as gender and infection status) were summarized as frequencies and percentages. For group comparisons or associations, Pearson’s chi-square test was used to compare categorical variables between groups. A *P*-value < 0.05 was considered the threshold for statistical significance. Data was verified by cross-referencing the hard copy forms and using Health Mapper software to confirm the survey site coordinates. To minimize bias, completed data forms were exchanged amongst team leaders for review. Discrepancies identified were resolved by the team leads during data validation meetings.

### Ethical consideration

The TAS is an integral component of the LF elimination programme and serves to evaluate the impact of MDA efforts. As such, formal ethical clearance was not required for this assessment. Nevertheless, ethical principles were strictly upheld throughout the study. Informed consent was obtained from both the participating schools and the parents or guardians of the children who took part in the assessment. The purpose of the survey was explained, participation was voluntary, and the children had the right to opt out of the survey at any point. If a child test positive and the second test confirms the positive result, the child is referred to be treated with ivermectin and albendazole, the caregivers are also notified and the case is reported to the programme for decision-making.

## Results

### Participant enrolment and demographic profile

A total of 6448 school-aged children were enumerated across the four evaluation units of Nigeria’s FCT. Participation exceeded the programme’s target sample size of 6128 by 5%, reflecting oversampling to compensate for potential absentees. Boys comprised 53% (*n* = 3417) and girls 47% (*n* = 3031) of the cohort; the sex ratio did not differ significantly across EUs. All surveys were conducted in 133 primary schools selected with the WHO school-based TAS protocol. TAS 1 was implemented in Gwagwalada and Bwari EUs, while TAS 2 was conducted in Abaji and Kuje EUs.

The numbers of pupils targeted, tested, and refusing participation, along with the counts of positive and invalid results for TAS 1 and TAS 2 are summarized in Tables 2 and 3, respectively.

**Table 2:** Result of TAS 1 conducted in Gwagwalada and Bwari EUs highlighting number of pupils tested, valid samples and refusals

EU	Area council	Target sample size	Pupils tested	Valid samples	Refusals	Number of schools survyed	Positives	Invalid tests	Critical cut-off
EU1	Gwagwalada	1540	1608	1597	11	30	0	0	18
EU2	Bwari	1552	1640	1606	34	30	0	0	18

*EU* Evaluation unit; *TAS* Transmission assessment survey

**Table 3:** Summary of TAS 2 results in 2 EUs

EU	Area council	Target sample size	Pupils tested	Valid sample	Refusals	Number of schools survyed	Positives	Invalid tests	Critical cut-off
EU1	Abaji	1532	1588	1568	13	36	1	7	18
EU2	Kuje	1532	1612	1597	10	37	0	5	18

*EU* Evaluation unit; *TAS *Transmission assessment survey

In TAS 1 (Table 2), 3248 children were tested across the two EUs (Gwagwalada and Bwari). Of these, 3203 samples were valid, and no positive cases were detected. No FTS-positive children were detected [observed prevalence 0%; 95% confidence interval (*CI)*: 0–0.09%], keeping each EU well below the critical threshold of 18 positives required to fail TAS 1. The spatial distribution of all 60 sampled schools showing no site recorded an antigen-positive pupil is shown in Fig. 4.

**Fig. 5:**
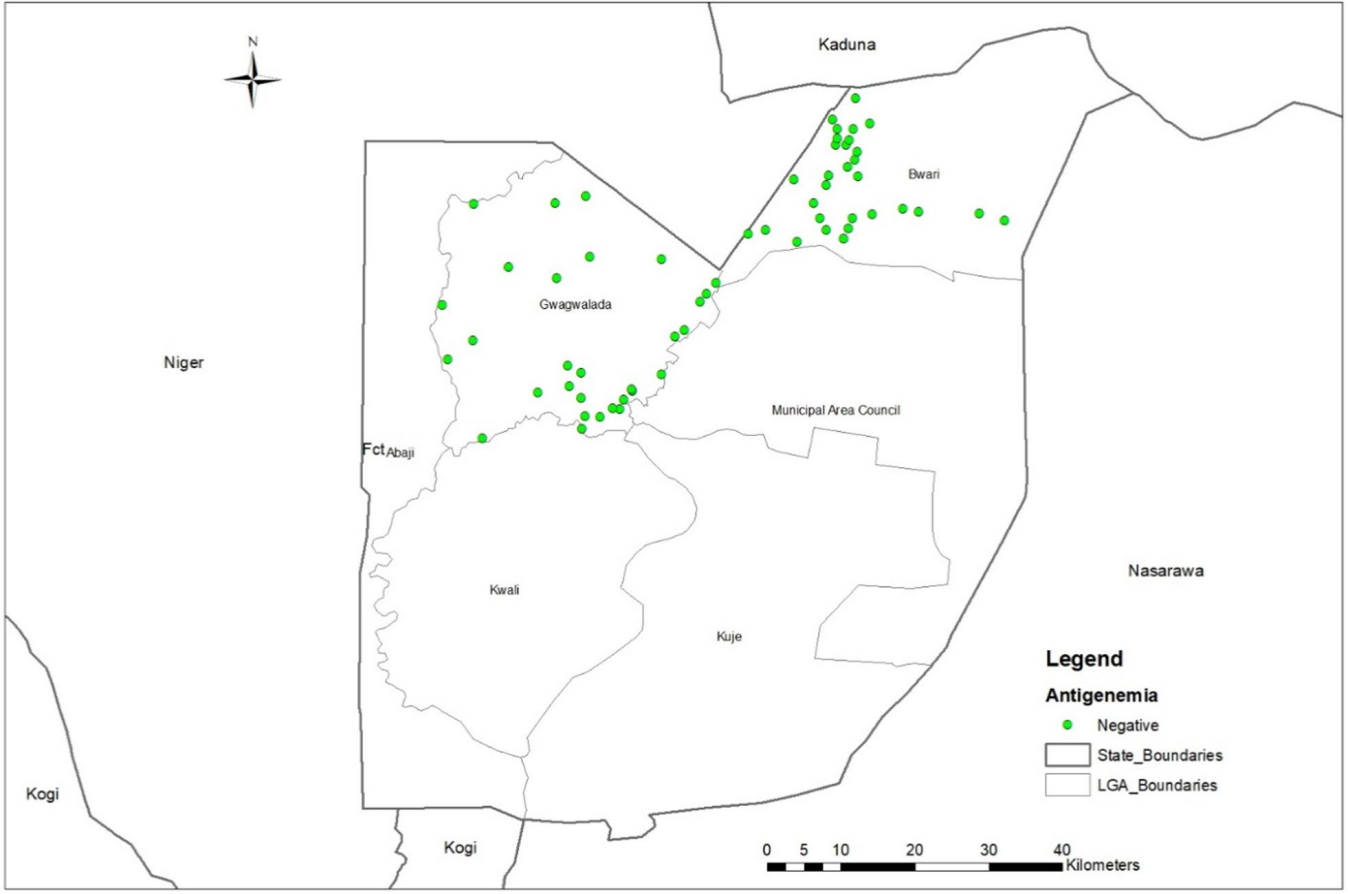
Map of FCT showing data collection sites for TAS 1 in Bwarii and Gwawalada EUs. Abbreviations: *FCT* Federal Capital Territory; *EU* Evaluation unit; *TAS* Transmission assessment survey

**Fig. 6:**
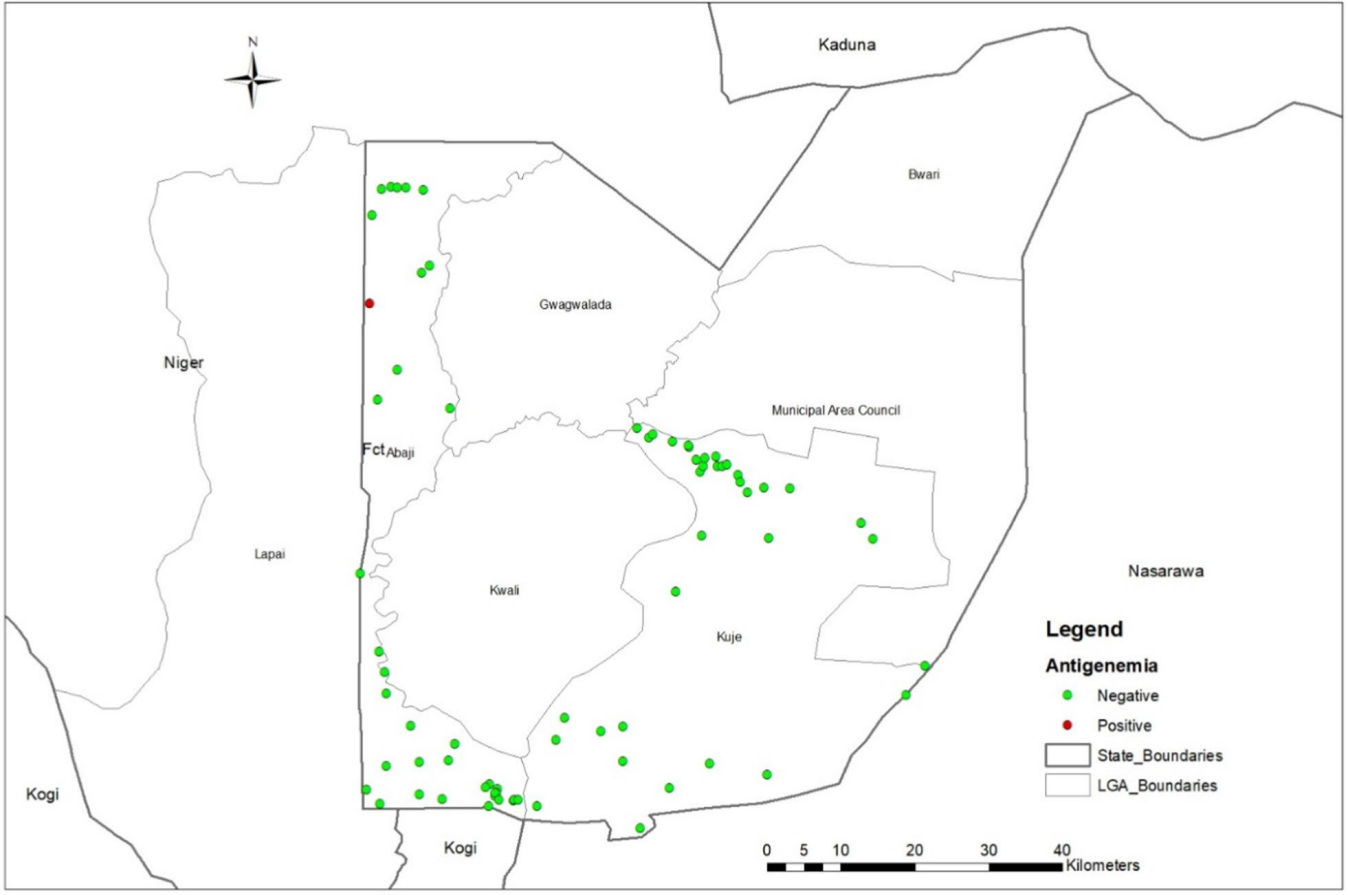
Map of FCT showing data collection sites for TAS 2 in Abaji and Kuje EUs and highlighting the sight where the positive case was recorded. Abbreviations: *FCT* Federal Capital Territory; *EU* Evaluation unit; *TAS* Transmission assessment survey

In TAS 2 (Table 3), 3200 children were tested in Abaji and Kuje, of which 3165 samples were valid. Only one positive case was recorded (in Abaji), which remained well below the critical cut-off. No positive cases were identified in Kuje. Figure 5 below also shows TAS 2 sites and the site where a positive case was recorded in Abaji EU (Fig. 6).

## Discussion

TAS are critical tools for monitoring the progress of LF elimination programmes. The findings from these surveys indicate that LF transmission in the FCT has been suppressed to a level that it is no more a public health problem. In the TAS 1 conducted in Bwari and Gwagwalada, no antigen-positive children were detected, while the result of TAS 2 conducted in Abaji and Kuje found only a single positive child (in Abaji). All EUs were well below the WHO critical cut-off thresholds for transmission. These results demonstrate that the criteria for stopping MDA have been met in the surveyed area [10]. Prevalence has declined dramatically from baseline (e.g. antigenemia in Abaji dropped from ~ 4% pre-MDA to ~ 0.1% in TAS 2, and Kuje from ~ 1% to 0%) [14]. Such pronounced reductions are consistent with reports from other LF-endemic settings—for instance, studies in Kenya, Benin, Togo and Egypt, have all documented significant declines in infection prevalence and intensity after 5–8 years of effective MDA [16–19]

The absence of positive cases in over three thousand samples collected and analysed from Bwari and Gwagwalada EUs provides evidence that LF transmission has been interrupted in these areas, hence, these areas meet the WHO criteria or stopping annual MDA [20]. This result is consistent with previous studies in other LF endemic regions, which have demonstrated that sustained high MDA coverage, coupled with comprehensive health education can lead to the interruption of LF transmission [16, 21–23]. Additionally, the results align with similar findings in countries such as Togo and Sri Lanka, where consistent MDA implementation and robust surveillance systems have led to the successful elimination of LF as a public health problem [23, 24].

The result of TAS 2 also demonstrates that Abaji and Kuje EUs have maintained the interruption of LF transmission status since 2021 when annual MDA was stopped. Although, one positive case was recorded in Abaji EU, LF transmission in the EU remains below the WHO recommended critical threshold for LF elimination as a public health problem (typically 18 positives in 1550 samples) [9], thus maintaining its status of interruption of LF transmission. However, targeted MDA will be conducted in the area where the positive case was recorded. This finding underscores the fact that even where prevalence has fallen below critical cutoffs, focal hotspots may persist due to low MDA coverage, localized vector abundance, or community-specific barriers to intervention uptake [6, 25, 26]. Similarly large-scale TAS implementations in Ethiopia and post elimination surveillance in Ghana have revealed pockets of persistent transmission despite overall progress, emphasizing the importance of geographically focused surveillance and response after assessment as this [5, 27, 28]. The single positive identified in Abaji illuminates a common challenge observed in countries like Tanzania and Ghana highlighting that those residual microfoci can reignite transmission if post-MDA surveillance and vector control measures are relaxed prematurely [28, 29].

The result of TAS 1 and TAS 2 highlight three critical insights. First, repeated rounds of MDA can significantly lower LF prevalence, thereby meeting WHO criteria for passing TAS 1 and stopping MDA [10, 11]. Second, even as LF transmission appears interrupted in certain localities, residual foci such as the one detected in Abaji underscore the need for continued surveillance and potentially targeted “mop-up” efforts [5, 30]. Furthermore, although the WHO-recommended filarial test strip is recognized as a reliable diagnostic tool, its sensitivity may wane in low-transmission settings, underscoring the potential value of molecular xenomonitoring to identify hidden reservoirs of infection [31].

Beyond epidemiological outcomes, the low refusal rates, as reported in these assessments, this highlights strong community acceptance of LF control activities and underscores how effective community engagement shapes community participation [32] and may indicate acceptance of similar public health interventions and research [33]. Additionally, as Nigeria’s capital city, FCT experiences high migration, it also shares boundaries with areas that have suboptimal LF MDA coverages hence, is important to note that the risk of recrudescence remains, particularly in areas like FCT which has high population mobility [34–36]. FCT is mostly urban surrounded with peri urban areas where treatment compliance could be challenging as reported in Kano State, Nigeria [37], and Tanzania [38]. As FCT proceeds to LF surveillance, lessons from the American Samoa could be adapted where alternative post‐MDA surveillance strategies leveraged on integration with other disease or public health initiatives, along with opportunistic screening of large population groups [39].

It is also important to note that the survey focused on children who were enrolled in selected schools. As documented in similar studies in rural Ethiopia and Uganda, out-of-school children or migratory populations may represent higher-risk groups that remain under-sampled in school-based surveys [34, 35]. Thus, targeted community surveys or integration with other NTDs platforms could strengthen surveillance and help identify potential for transmission in these overlooked populations.

Although TAS are widely endorsed for post-MDA verification, the child-centred design introduces blind spots that can mask lingering transmission. By sampling only 6–7-year-olds, TAS may miss low-density infections persisting in adults, out-of-school children, or highly mobile groups limitations documented in American Samoa, where concurrent community surveys uncovered > 100 infections despite a passing TAS[39]. Moreover, the FCT evaluation relied solely on antigen detection with FTS; while more sensitive than the older ICT card, FTS still fails to detect a small share of very low-intensity infections [40]. The absence of confirmatory microfilaria tests, entomological xenomonitoring, or antibody assays means that low-grade transmission could have gone undetected, an issue highlighted in Ghana and American Samoa, where molecular and vector data have revealed residual hotspots after apparently successful TAS rounds [41, 42]. Thus, the study’s zero-prevalence finding in children cannot be equated unequivocally with complete interruption of transmission.

Contextual constraints further limit generalisability. The two FCT councils surveyed were relatively low-endemic and benefited from strong partner support, in contrast to several high-burden Nigerian districts still struggling with insecurity, urban complexity, or sub-optimal coverage [37, 43]. Unmeasured factors such as population mobility across state borders, vector-control activities such as insecticide-treated-net use, or COVID-19-related programme disruptions could influence long-term outcomes but were not assessed. Taken together, these methodological and contextual limitations underscore that a successful TAS 1/TAS 2 represents a critical milestone rather than definitive proof of elimination, and they justify continued surveillance to safeguard against recrudescence.

The FCT provides evidence that with sustained programme rigor, elimination of lymphatic filariasis as a public-health problem is attainable in Nigeria. The four LF endemic area councils in the FCT have already stopped MDA, demonstrating to other states that the 2030 WHO targets are realistic [44]. Replicating this progress elsewhere will require innovative, context-specific approach for harder-to-reach, increasingly urbanised areas: targeted social-mobilisation in city neighbourhoods, closer collaboration with municipal health structures, and integration of LF drug delivery into broader campaigns such as malaria or COVID-19 outreach [45]. Where epidemiologically appropriate, programmes could also fast-track parasite clearance by introducing triple-drug IDA therapy, as endorsed by recent WHO guidance [46].

Equally important is safeguarding these gains. WHO calls for follow-up TAS surveys at 2–3 and 4–6 years after stopping MDA to detect any recrudescence early [10]; FCT should adhere to this timetable while maintaining community vigilance for new cases. The programme should also scale up morbidity-management and disability-prevention services hydrocele surgery and lymphoedema care to satisfy elimination validation criteria and meet patient needs [20]. Evidence from Malawi and other settings shows that such interventions markedly improve quality of life and household economics, breaking the poverty loop associated with LF [47, 48].

## Conclusions

Successive TAS have now confirmed that all four LF endemic EUs within the FCT have moved from active transmission to sustained interruption of LF. This trajectory from baseline endemicity, through high coverage mass MDA, intensive community mobilisation, and post-MDA surveillance, shows that WHO-recommended strategies, when adapted to local contexts, can eliminate LF even in densely populated urban and peri-urban settings. However, the detection of a single antigen-positive child in Abaji underscores the importance of targeted “mop-up” treatment and the future use of molecular and entomological tools to prevent recrudescence.

Overall, TAS 1 results from Gwagwalada and Bwari verify transmission interruption, while the TAS 2 findings from Abaji and Kuje demonstrate the consolidation of those gains, keeping all EUs well within WHO elimination thresholds. To safeguard this progress, the programme must maintain targeted surveillance, strengthen school- and community-based health education, and adopt more sensitive diagnostics and integrated vector management—especially to reach out-of-school or migratory children and detect very low-level infection[49, 50]. With these measures, FCT will sustain elimination and provide a blueprint for other high-burden settings approaching the LF end game.

## Data Availability

The datasets during and/or analysed during the current study available from the corresponding author on reasonable request.

## Abbreviations

GPELF: Global Programme to Eliminate Lymphatic Filariasis Strategy
EU: Evaluation unit
FCT: Federal Capital Territory
FTS: Filarial Test Strip
IVM: Integrated vector management
IQR: Interquartile range
LF: Lymphatic filariasis
LGA: Local government area
MDA: Mass drug administration
MMDP: Morbidity management and disability prevention
NTD: Neglected tropical disease
Pre-TAS: Pre-transmission assessment survey
SD: Standard deviation
TAS: Transmission assessment survey
VC: Vector control
